# Impact of educational instruction on medical student performance in simulation patient

**DOI:** 10.5116/ijme.62a5.96bf

**Published:** 2022-06-23

**Authors:** Logan D. Glosser, Conner V. Lombardi, Wade A. Hopper, Yixing Chen, Alexander N. Young, Elliott Oberneder, Sprio Veria, Benjamin A. Talbot, Shirley M. Bodi, Coral D. Matus

**Affiliations:** 1 Department of Medical Education, University of Toledo College of Medicine and Life Sciences, USA; 2 Department of Medical Education, Edward Via College of Osteopathic Medicine, USA; 3 Lloyd A. Jacobs Interprofessional Immersive Simulation Center, USA; 4 Department of Family Medicine University of Toledo College of Medicine and Life Sciences, USA

**Keywords:** Simulated patient encounters, clinical reasoning, medical education, educational intervention, curriculum development

## Abstract

**Objectives:**

This study aimed to evaluate the effects, and timing of, a video educational intervention on medical student performance in manikin-based simulation patient encounters.

**Methods:**

This prospective mixed-methods study was conducted as part of the University of Toledo College of Medicine and Life Sciences undergraduate medical curriculum. One hundred sixty-six students second-year students participated in two simulations on a single day in September 2021. A 7-minute video intervention outlining the clinical diagnostic approach to pulmonary complaints was implemented. Students were randomized into 32 groups which were divided into two cohorts. One received the video prior to simulation-1 (n=83) and the other between simulation-1 and simulation-2 (n=83). Each simulation was recorded and assessed using a 44-point standardized checklist. Comparative analysis to determine differences in performance scores was performed using independent t-tests and paired t-tests.

**Results:**

Independent t-tests revealed the video-prior cohort performed better in simulation-1 (t_(30)_= 2.27, p= .03), however in simulation-2 no significant difference was observed between the cohorts. Paired t-test analysis revealed the video-between cohort had significant improvement from simulation-1 to simulation-2 (t_(15)_= 3.06, p = .01); no significant difference was found for the video-prior cohort. Less prompting was seen in simulation-2 among both the video-prior (t_(15)_= –2.83, p= .01) and video-between cohorts (t_(15)_= –2.18, p= .04).

**Conclusions:**

Simulation training, and targeted educational interventions, facilitate medical students to become clinically competent practitioners. Our findings indicate that guided video instruction advances students' clinical performance greater than learning through simulation alone. To confirm these findings, similar investigations in other clinical training exercises should be considered.

## Introduction

Healthcare is a profession that is continually evolving and, along with it, medical student education. As disease patterns, diagnostic tools, and the healthcare landscape have changed, so too have the current medical curricula leading to models that focus on achieving various competencies to assess the readiness of future physicians.^1^ One such change has been an increasing utilization of manikin-based simulation patient encounters (SPEs) to facilitate medical student acquisition of clinical skills.^2^

Despite sophisticated curriculum design and complex teaching methods utilized in medical student education, disparities exist among newly graduated physicians and their ability to effectively transfer basic science knowledge to clinical practice^3^^,^^4^ There are many challenges facing medical educators in helping students attain autonomous learning and critical thinking skills.^5^^,^^6^ Critical thinking skills allow medical students to triage clinical scenarios, respond promptly, and make reasonable clinical decisions to provide quality patient care.^6^ With modern technology rapidly integrating into health care, it can feel as though doctors are akin to a computer, expected to analyze an immense amount of data to identify symptoms, diagnose disease, and compute the appropriate management.^7^

To develop these skills, medical education has become largely focused on the vertical integration (VI) method, defined as the gradual transition from the classroom to clinical environments.^8^^,^^9^ The VI method bridges students from acquiring knowledge to implementation and critical thinking in clinical practice.^8^^,^^9^ This method has demonstrated increased learning retention, although difficult to achieve.^10^ Moreover, there are numerous strategies available for educators to facilitate this transformation from classroom learning to application in practice. These strategies include the use of standardized patients, exposure to patient care through observation, and increasingly manikin-based SPEs.^11^^-^^13^ These strategies are typically used in combination, such that knowledge acquisition occurs with concepts presented over time and occur in different learning environments.^8^^,^^10^

Although the use of manikin-based SPEs have been adopted by most medical schools in the United States, there is significant heterogeneity by which such exercises are conducted and evaluated. Currently, no standardized guidelines exist for designing simulation experiences to optimize medical student learning.^14^^,^^15^ It is unknown whether guided educational instruction impacts medical student simulation performance and learning. However, prior evidence suggests that medical students value such clinical simulations and see them as a helpful learning modality.^16^^-^^19^

Medical education, and the curriculum that guides it, require continual evaluation to adapt to change.^20^ This includes a rigorous evaluation of simulation-based education especially given the advances and the adoption of this type of learning. As such, the objective of this study was to determine the impact, and timing of, an educational video on medical student performance in manikin-based simulation. The main research questions were:

    ·  Does peri-simulation education improve medical student clinical performance during the encounter?

    ·  Does the timing of such education alter medical student performance, and learning, in simulation patient encounters?

    ·  How do students perceive simulation training, and peri-simulation education, to impact their clinical abilities?

## Methods

### Study design and participants

All second-year medical students attending the University of Toledo College of Medicine and Life Sciences were eligible to participate in the study. The trial was explained in detail to the students, and they were assured of the anonymity and confidentiality of personal information for all responses. The University of Toledo Institutional Review Board gave approval for the study. A total of 176 medical students were eligible to participate. Ten students were unable to participate due to infection with COVID-19, leaving a total of 166 students who participated in the SPE's.

We undertook a mixed-methods study to determine the impact of a peri-simulation educational video on student performance and learning in two simulations. A cross-over type design for the video intervention was implemented, in which students either received the video before the first SPE (SPE-1) or the video between SPE-1 and the second SPE (SPE-2). A pre-test multiple-choice questionnaire (MCQ) was administered prior to SPE-1, and a post-test MCQ was administered after SPE-2, followed by a voluntary feedback survey and a debriefing of the experience with a faculty physician. The SPE scenarios encompassed a patient with asthma and a patient with chronic obstructive pulmonary disease (COPD).

**Figure 1:**
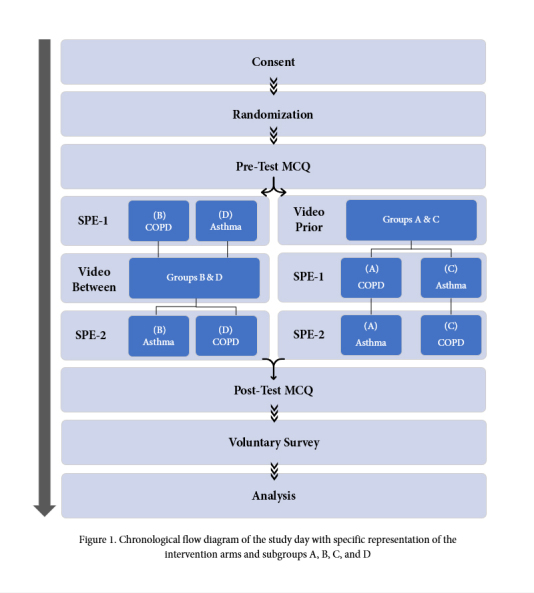
Chronological flow diagram of the study day with specific representation of the intervention arms and subgroups A, B, C, and D

A two-stage randomization strategy was implemented, as shown in Figure 1. First, students were randomized into 32 groups of approximately equal size (5–6 students each) by faculty independent of the research team. Assignment of students and the randomization process was concealed from the students and the research team. The 32 groups were then randomized into four subgroups (A, B, C, D). The four subgroups (A, B, C, and D) had a pre-randomization of the location of the simulation room and the SPE scenario.

Subgroups A and C both received video interventions before the first simulation scenarios and rotated thru the same environment: room, medical manikin, faculty, and simulation operator. Subgroup A was given the COPD simulation scenario first, followed by the asthma scenario and subgroup C was given the asthma simulation scenario first, followed by the COPD scenario. Subgroups B and D both received video interventions between the simulation scenarios and rotated thru the same environment: room, medical manikin, faculty, and simulation operator. Subgroup B was given the COPD simulation scenario first, followed by the asthma scenario and subgroup D was given the asthma simulation scenario first, followed by the COPD scenario.

There was a total of four operators running the SPE environments. The operators ran the same case in the same room and manakin for the duration of the study. The operators were given a standardized training session for their respective cases to keep the language and overall atmosphere the same for each group.

### Simulation scenarios

The two SPE scenarios and the information the students were expected to obtain were developed by faculty, independent of the research team, as part of the standard undergraduate medical education curriculum. The SPE's were a formative, non-graded group activity designed as an opportunity for medical student learning.

The students were given the following information on a display screen prior to starting the SPE: "You will have 15 minutes to conduct a focused history and physical exam. You will present the case afterwards as you will do on your clerkship rotations."

The COPD case represented a 61-year-old male, one pack/day smoker since he was 15 years old, with worsening dyspnea over the past two to three days, acutely worse the morning of the presentation. Relevant high-yield objective information given to the students when prompted included a temperature of 37.1 °C, heart rate of 124 beats per min, respiratory rate of 28 breaths per min, blood pressure 149/94, pulse oximetry oxygen saturation of 84%, forced expiratory volume in one sec to force vital capacity ratio 60% of expected, brain natriuretic peptide level of 88 pg/mL, initial arterial blood gas pH of 7.3, pCO2 of 55 mmHg, pO2 of 55 mmHg, bicarbonate of 26 mmHg, and imaging consistent with acute COPD exacerbation.

The asthma case represented a 10-year-old boy with dyspnea after playing outside. Students were notified that he was visibly distressed in the waiting room, where he was bent over with his hands on his knees. Relevant high-yield objective information given to the students when prompted included a temperature of 37 °C, blood pressure of 104/68 mmHg, heart rate of 110 beats per min, respiratory rate of 30 breaths per min, pulse oximetry oxygen saturation of 84%, forced expiratory volume in one sec to force vital capacity ratio 65% of expected, pulmonary function test after beta-agonist therapy 85% of expected, initial arterial blood gas pH of 7.37, pCO2 of 55 mmHg, pO2 of 65 mmHg, bicarbonate of 22 mmHg, and imaging consistent with acute asthma exacerbation.

### Instruments

Simulation performance scores were calculated using separate standardized checklists for the COPD and asthma scenarios, respectively, as shown in Table 1. The checklists were modified versions of those used for the graded objective structured clinical examinations performed as part of the standard undergraduate medical education curriculum.

**Table 1:** Checklist scoring system for the Asthma and COPD cases

Asthma		COPD	
Category (max points)	Points	Category (max points)	Points
HPI (19)		HPI (19)	
Patient name	1	Patient name	1
Chief complaint	1	Chief complaint	1
Location/radiation	1	Location/radiation	1
Quantity/severity	1	Quantity/severity	1
Timing (Onset/frequency/duration)	1	Timing (Onset/frequency/duration)	1
Setting in which it occurs	1	Setting in which it occurs	1
Exacerbating factors	1	Exacerbating factors	1
Remitting factors	1	Remitting factors	1
Associated symptoms	1	Associated symptoms	1
Patient perspective	1	Patient perspective	1
Medications	1	Medications/Lisinopril	1
Allergies (agent & reaction)	1	Allergies (agent & reaction)	1
Tobacco use/Exposure	1	Tobacco use/smoking	1
Alcohol use	1	Alcohol use	1
Illicit drug (type, quantity/frequency)	1	Illicit drug (type, quantity/frequency)	1
ROS (10 categories for a point)	1	ROS (10 categories for a point)	1
Family History	1	Family History	1
Vaccinations	1	Vaccinations	1
Past Surgical Hx	1	Past surgical hx	1
Physical exam (11)		Physical exam (11)	
Auscultate	1	Auscultate	1
Identified wheezing	1	Identified wheezing	1
Palpate/percuss	1	Palpate/percuss	1
Look to assess symmetry/contour	1	Look to assess symmetry/contour	1
Assessed airway	1	Assessed airway	1
Repositioning	1	Repositioning	1
Heart rate	1	Heart rate	1
RR	1	RR	1
Temperature	1	Temperature	1
BP	1	BP	1
o2 sat	1	o2 sat	1
A/P/I (14)		A/P/I (14)	
Ask for CXR	1	Ask for CXR	1
Identified pulmonary infiltrates	1	Identified barrel-chest/enlarged lung fields	1
Ask for ABG	1	Ask for ABG	1
Identified respiratory acidosis	1	Identified respiratory acidosis	1
Ask for CBC	1	Ask for CBC	1
Eosinophilia	1	Ask for spirometry or PFT	1
Ask for spirometry or PFT	1	Identified Obstructive pattern	1
Identified Obstructive pattern	1	Ask for EKG	1
Ask for CMP/BMP	1	Ask for CMP	1
Identified respiratory alkalosis / decreased co2	1	Identified respiratory acidosis / elevated co2	1
Administered Non-rebreather	1	Administered Non-rebreather	1
Administered Nasal cannula	1	Administered Nasal cannula	1
Administered Albuterol/b2 agonist	1	Administered Albuterol/b2 agonist	1
Administered Steroid	1	Administered Steroid	1
Prompting	*	Prompting	*

Note: HPI = History of presenting illness; A/P/I = assessment/plan/intervention; * = no maximum number of promptings. The maximum number of points available for scoring was 44 for both the COPD and asthma scenarios.

Two faculty physicians performed checklist modifications for the SPEs. It encompassed key aspects of the history of presenting illness (HPI), physical exam, assessment/plan/interventions (A/P/I), and the number of times the facilitator had to prompt students to keep the sim moving forward. There was a total of 44 checklist items for SPEs, with each checklist item worth 1 point.

Individual baseline student knowledge was assessed by completing a multiple-choice question (MCQ) pre-test examination before SPE-1, as shown in Appendix 1. At the conclusion of SPE-2, students were re-assessed using a post-test MCQ, which consisted of the same ten questions and answers (ranging from two to five possible answers) as the pre-test. The MCQ points per question were weighted based on difficulty as pre-assigned by a faculty physician on the study personnel. There were three questions weighted to be worth 2 points, one question worth 3 points, and the remaining six questions each worth 1 point, totaling a maximum of 15 points available. The MCQ examination was intended to measure student learning separate from the dimension of clinical performance. Expert physicians verified the content validity of the MCQ. However, no statistical certification of MCQ reliability was performed.

Students had the option to voluntarily complete an anonymous 5-point Likert Scale feedback questionnaire at the conclusion of the MCQ post-test. The questionnaire was composed of three questions intended to gather students' perspectives on the impact of the intervention, debriefing, and overall simulation training experience on their learning.

### Data collection methods

Two blinded independent evaluators viewed video recordings of the SPEs. Both reviewers were 4th-year medical students who were trained independently by a faculty to ensure the accuracy of the assessment. The reviewers were blinded from the intervention arms of the groups were a part of, and they were blinded from whether the group was in SPE-1 or SPE-2. The evaluators recorded whether the students performed the checklist items for the respective Asthma and COPD scenarios. A third independent reviewer compiled the two reviewers' checklists and found no instances in which the independent reviewers differed in their scoring. The MCQ pre-and post-tests, along with the feedback questionnaire, were performed using an online platform which recorded each student's responses. Faculty placed the students' scores into their respective groups such that individual students' scores remained deidentified, and performance scores could not be traced back to the individual name of the student.

### Statistical analysis

The primary objective was to compare the performance effect as measured by an adapted analysis of student performance in a group setting of simulated patient encounters. The hypothesis that the Intervention = 0 was tested by means of student paired t-tests and independent t-tests. The scores were expressed as means plus or minus standard deviations (SD). The mean and SD of the within-group difference captures the treatment effect and the paired nature of the design; these were used as the basis for constructing the difference between the means confidence intervals and hypothesis testing. This analysis approach made two main assumptions (in addition to the normality assumption for the student's paired t-test): no period effect and no intervention-period interaction. Secondary analysis was performed to compare the sub-categorization of the scoring checklist, compare the multiple-choice questionnaire pre-test and post-test, and compare the COPD versus asthma performance scores from SPE-1 and SPE-2. All statistical tests were two-sided, and a p-value ≤ .05 indicated statistical significance. All statistical tests were performed using Microsoft Excel.

## Results

One hundred sixty-six students participated in the study. A total of 64 SPEs were assessed (two SPEs for each of the 32 groups). The separate intervention arms' mean simulation totals and sub-categorical scores of SPE-1 and SPE-2 are shown in Table 2. A comparison of the video prior versus the video between total and sub-categorical scores in SPE-1 and SPE-2 are shown in Table 3.

A significant difference was observed when comparing the mean performance scores the two intervention arms in SPE-1, with the video prior cohort performing better with a mean of 23.81 (SD= 3.41) versus 21.06 (SD= 3.41) on independent t-test (t_(__30)_= 2.27, p = .029). The video prior cohort scored higher in the HPI subcategory of SPE-1 with a mean of 11.25 (SD= 2.72) versus 9.31 (SD= 2.15) among the video between cohort (t_(__30)_= 2.23, p = .033). The video prior cohort had a lower mean number of times prompting in SPE-1 of 2.06 (SD= 0.93) compared to 3.68 (SD= 2.08) among the video between (t_(__30)_= –2.84, p = .007).

In SPE-2 no significant difference was found between the total performance scores. There was a significant difference between the number of times prompted, with the prior video cohort requiring a lower number of times prompted with a mean of 1.06 (SD= 0.77) versus 2.31 (SD= 1.25) for the video between cohort (t_(__30)_= –3.40, p = .001).

No significant difference among the video prior cohort was observed when analyzing the scores using paired t-tests of SPE-1 vs SPE-2 (t_(__15)_= –1.18, p = .257). A significant difference was seen between the SPE-1 and SPE-2 scores of the video between cohort (t_(__15)_= 3.06, p = .008), mainly attributed to a significant difference in the HPI sub-categorical score (t_(15)_= 4.50, p =.001). A significant difference was seen in the number of times prompted for both the video prior and the video between cohorts (respectively t_(__15)_= –2.83, p = .01, and t_(15)_= –2.18, p = .04).

**Table 2:** Mean simulation performance total and sub-categorical scores by intervention arm

Categories	N	Max score	SPE-1 mean (SD)	SPE-2 mean (SD)	Paired t-test t score	p-value	95% CI	
							LL	UL
Video Prior								
Total	16	44	23.81 (3.41)	22.37 (4.06)	t_(__15) _= –1.18	.257	–1.27	4.15
HPI		19	11.25 (2.72)	10.25 (3.43)	t_(__15) _= –1.35	.197	–1.23	3.23
Physical exam		11	5.87 (1.78)	5.12 (1.62)	t_(__15) _= –1.12	.279	–0.48	1.98
A/P/I		14	6.68 (2.35)	6.81 (1.93)	t_(__15) _= 0.16	.877	–1.68	1.42
Prompting		*	2.06 (0.93)	1.06 (0.77)	t_(__15) _= –2.83	.012	.38	1.62
Video B/T								
Total	16	44	21.06 (3.41)	24.62 (4.20)	t_(__15) _= 3.06	.008	–6.32	–0.80
HPI		19	9.31 (2.15)	12.43 (2.87)	t_(__15) _= 4.50	0.001	–4.95	–1.30
Physical exam		11	5.12 (1.66)	5.43 (2.12)	t_(__15) _= 0.55	.590	–1.68	1.06
A/P/I		14	6.62 (2.21)	6.75 (1.94)	t_(__15) _= 0.22	.826	–1.63	1.37
Prompting		*	3.68 (2.08)	2.31 (1.25)	t_(__15) _= –2.18	.045	0.13	2.61
Combined Video Prior + Video B/T								
Total	32	44	22.43 (3.63)	23.50 (4.22)	t_(__31)_= 1.13	.269	–2.41	0.27

Note: HPI = History of presenting illness; A/P/I = assessment/plan/intervention; *= no maximum number of promptings; SPE-1 = first simulated patient encounter; SPE-2 = second simulated patient encounter; CI = confidence interval; LL = lower limit; UL = upper limit. The 95% CI is reported for the difference between the means.

**Table 3:** Comparison of the intervention arms mean total and sub-categorical performance scores

Categories	N	Max score	SPE-1 mean (SD)	SPE-2 mean (SD)	t-test t score	p-value	95% CI	
							LL	UL
Total Score		44						
Video Prior	16		23.81 (3.41)		t_(__30) _= 2.27	.029	0.29	5.21
Video B/T	16		21.06 (3.41)					
Video Prior	16			22.37 (4.06)	t_(__30) _= –1.53	.134	–5.23	0.73
Video B/T	16			24.62 (4.20)				
HPI		19						
Video Prior	16		11.25 (2.72)		t_(__30) _= 2.23	.033	0.17	3.71
Video B/T	16		9.31 (2.15)					
Video Prior	16			10.25 (3.43)	t_(__30) _= –1.95	.060	–4.46	0.10
Video B/T	16			12.43(2.87)				
Physical Exam		11						
Video Prior	16		5.87 (1.78)		t_(__30) _= 1.23	.228	–0.49	1.99
Video B/T	16		5.12 (1.66)					
Video Prior	16			5.12 (1.62)	t_(__30) _= –0.47	.644	–1.67	1.05
Video B/T	16			5.43(2.12)				
A/P/I		14						
Video Prior	16		6.68 (2.35)		t_(__30) _= 0.08	.938	–1.59	1.71
Video B/T	16		6.62 (2.21)					
Video Prior	16			6.81 (1.93)	t_(__30) _= 0.09	.928	–1.34	1.46
Video B/T	16			6.75 (1.94)				
Prompting		*						
Video Prior	16		2.06 (0.93)		t_(__30) _= –2.84	.007	–2.78	–0.46
Video B/T	16		3.68 (2.08)					
Video Prior	16			1.06 (0.77)	t_(__30) _= –3.40	.001	–2.00	–0.50
Video B/T	16			2.31 (1.25)				

Note: Video B/T = video between; * = no maximum number of prompting; HPI = History of presenting illness; A/P/I = assessment/plan/intervention; SPE-1 = first simulated patient encounter; SPE-2 = second simulated patient encounter; CI = confidence interval; LL = lower limit; UL = upper limit. The 95% CI is reported for the difference between the means.

To ensure a true difference was observed, a sub-analysis of the scenarios was performed. Independent t-tests showed no significant difference between the SPE-1 scores when analyzing asthma vs COPD scenarios nor the SPE-2 scores of asthma vs COPD scenarios (Appendix 2). Additionally, no significant differences were shown between the SPE-1 and SPE-2 scores of the asthma scenario alone or the COPD scenario alone.

For the sub-analysis of the MCQ performance results, the distribution of measured baseline variables was balanced between the two intervention arms. The mean MCQ pre-and post-test performance scores are shown in Table 4. A significant difference was found when assessing the MCQ pre-and post-test scores of the total cohort, with a mean MCQ pre-test score of 11.33 (SD= 0.93) to a mean of 13.74 (SD= 0.67) on the post-test (t_(__31)_= 13.23, p = .001). This difference was seen when separately assessing the video prior and video between cohorts mean pre-and post-test MCQ scores (respectively t_(__15)_= 10.14,  p = .001, and t_(15)_= 8.44, p = .001).

**Table 4:** Mean multiple-choice questionnaire (MCQ) pre- and post-test performance scores

Groups	N	Pre-test mean (SD)	Post-test mean (SD)	Paired t-test t score	p-value	95% CI	
						LL	UL
Total	32	11.33 (0.93)	13.74 (0.67)	t_(__31) _= 13.23	.001	–2.81	–2.00
Video prior	16	11.28 (1.06)	13.76 (0.51)	t_(__15) _= 10.14	.001	–3.08	–1.88
Video B/T	16	11.38 (0.82)	13.71 (0.81)	t_(__15) _= 8.44	.001	–2.92	–1.74

Note: Video B/T = Video Between; CI = confidence interval; LL = lower limit; UL = upper limit. The 95% CI is reported for the difference between the means.

One hundred and twenty-three students completed the voluntary student feedback questionnaire. The majority of those who completed the survey (75%) of students indicated that they either agreed or strongly agreed that the educational intervention was beneficial to their learning. Contrarily, only a minimal (5%) of students indicated that they did not find the intervention to be beneficial. The three-question student feedback questionnaire using a 5-point Likert Scale, and the responses given are shown in Appendix 3.

## Discussion

Simulated Patient Encounters are becoming a mainstay of the medical school curriculum as medical educators continue to recognize the benefit of risk-free clinical skills practice that simulated clinical scenarios offer, especially to medical students in their pre-clinical years.^21^ The process of learning, especially for medical students, goes beyond memory recall as students need to learn how to demonstrate both competencies of knowledge and trust-worthy empathetic connections with patients.^22^ Physician empathy is not only a foundation of the physician-patient relationship, it also increases patient satisfaction,^23^ adherence to medical therapy,^24^ and improves patient outcomes.^25^ SPEs allow medical students the ability to practice their clinical skills, history and physical examinations, and medical knowledge in a simulated environment where they are encouraged to try and fail before they enter the clinical environment to work with real patients. Although several studies have investigated the effectiveness of SPEs in medical education, limited information exists on the impact and timing of guided peri-simulation education on student performance. In this study, our team investigated how a video educational intervention administered either before or between simulations affected student performance in respiratory SPEs.

### Impact of peri-simulation education

The cohort that had the educational intervention between SPE-1 and SPE-2 had a significant improvement in their performance in SPE-2. On further analysis, this improvement was mostly attributable to higher accuracy in the HPI subcategory. The improvement among the students with the educational intervention was most likely due to the intervention itself, as the analysis of all groups showed no significant difference between the performance on SPE-1 and SPE-2 (Table 2). When comparing the performance scores across the two SPE's for those who received the video intervention prior to SPE-1, there was no significant difference. This suggests the intervention had made the impact already, and that impact was retained in SPE-2.  Furthermore, this may indicate that the score improvement from the video intervention between cohort was not due to repeated exposure and practice.

The performance of the students in the SPEs showed the educational intervention had a positive impact on student performance. The students with the educational intervention prior to SPE-1 scored higher than those without the intervention, and although the educational intervention between-group scored higher on SPE-2, there was not a statistically significant difference between the intervention arms. This suggests that the intervention prior to the SPE-1 had a positive impact on student performance across both SPE-1 and SPE-2. When assessing the group performance by comparing the scores from COPD vs asthma, there was no significant result, suggesting that the cases themselves did not impact the group performance scoring assessment.

Both cohorts had a significant reduction in the number of times they needed to be prompted to continue progressing through the simulated patient encounters, which is most likely attributable to the experience of the simulation itself rather than the educational intervention. As previously mentioned, patient simulation is an experience where students are encouraged to try and fail. It often takes students an initial period to familiarize themselves with the simulation environment and be comfortable enough to progress through various portions of the simulation assertively. Thus, it is not surprising that students needed to be prompted less in SPE-2 as they were likely less apprehensive about the simulation experience in general.

Overall, both cohorts had similar baseline results from the pre-test multiple-choice questionnaire, which suggests that baseline knowledge did not impact the results. Furthermore, both cohorts had statistically significant improvement from the pre-test to post-test MCQ results. This lends to the fact that the exercise had a positive impact on student learning.

### Strengths and limitations

The major benefit of the cross-over design in this cohort is that it allowed within-group comparison, which would not have been possible in a conventional parallel-group design. In addition, this allowed all students to receive the educational intervention as part of the simulation experience. While a control group that did not receive the educational intervention in any respect would have been optimal to understand the exact impact of the educational intervention, regardless of its temporal placement, this was not ethically possible as every medical student needed to have the same amount of instruction during the SPE day.

There are several limitations to the study that we felt were worth noting. Our study used a crossover-type design for the intervention, rather than one group receiving the intervention and one no-intervention group. Although a true control group may provide a stronger argument, this design was used in an attempt to provide all the students with the same overall learning experience. Additionally, the simulated patient scenarios may have differed depending on the order in which the students' gathered information and asked questions. Although the operators of the case scenarios underwent training to standardize the experience, the presentation of the cases may have differed slightly between the groups from intrapersonal and interpersonal differences between the two operators running each simulation case. Despite simulation rooms being standardized for patient positioning and equipment necessary for the experience, there was a slight variation in the overall atmosphere. This difference included fixed structural variations of the rooms as they were built as either an operating room, a labor and delivery room, an emergency department room, or an intensive care unit room, which may have impacted how students approached the simulated patient. Finally, the SPEs in our study were formative, non-graded activities. As such, this may have impacted students' engagement in the SPEs and therefore their performance.

## Conclusions

Our study showed that a simulated patient encounter platform in combination with a clinical reasoning framework is an effective method that can be used in medical education. Integration of succinct learning objectives with educational interventions improved diagnostic assessment and rates of correct diagnosis. Learners showed simulation performance improvement directly following the educational intervention regardless of whether the intervention was delivered before or in-between exercises. Most participants found that the peri-simulation education was beneficial to their learning. Similar investigations in other medical student clinical training exercises should be explored to improve the learning process in manikin-based simulation patient encounters.

### Acknowledgements

We would like to give a special thanks to the following for their contributions to the study: Karla Dixon, the Lloyd A. Jacobs Interprofessional Immersive Simulation Center staff, and The University of Toledo College of Medicine and Life Sciences.

### Conflicts of Interest

The authors declare that they have no conflict of interest.

## Supplementary material

Supplementary file 1 Appendix 1. Multiple-Choice Questionnaire (MCQ) pre-test and post-test

Supplementary file 2 Appendix 2. Mean performance scores by COPD and Asthma case scenarios

Supplementary file 3 Appendix 3. Likert Scale student feedback questionnaire
