# Supplementary material for: Impact of educational instruction on medical student performance in simulation patient
Source: Int J Med Educ. 2022 Jun 23;13:158–70. doi: 10.5116/ijme.62a5.96bf (PMC9911140; doi:10.5116/ijme.62a5.96bf)
Supplement: Supplementary file 1 — Appendix 1. Multiple-Choice Questionnaire (MCQ) pre-test and post-test [file ijme-13-158-S1.pdf]

Appendix 1

Multiple-Choice Questionnaire (MCQ) pre-test and post-test

1. Alcohol and Smoking are documented under which subheading on a SOAP note? (1 Point)

*a. Social History
b. Review of Systems
c. History taking
d. Medications
e. Family History

2. During the physical exam, a patient randomly brings up that he has had difficulty starting to pee when he needs to go, you should document this under which portion of the SOAP note? (1 Point)

a. HPI - history of presenting illness
b. PMH - past medical history
c. SH - social history
*d. ROS - Review of Symptoms
e. Physical exam

3. If you measure that a patient is febrile at 38°C, you would place this in the _______ portion of the SOAP note. (1 Point)

a. Subjective
*b. Objective

4. The review of systems (ROS) should be documented under which portion of the SOAP note? (1 Point)

*a. Subjective
b. Objective
c. Assessment
d. Plan

5. What is the normal laboratory range of CO2 in mmHg on ABG? (2 Points)

a. 10-15
b. 15-20
c. 25-35
*d. 35-45

6. Asthma attacks demonstrate a(n) _____ FEV1/FVC ratio on PFTs and is classified as a(n) ______ lung disease. (3 Points)

a. Increased; Obstructive
b. Increased; Restrictive
*c. Decreased; Obstructive
d. Decrease; Restrictive

7. Which of the following medications is associated with causing a chronic cough? (2 Points)

a. Albuterol (B2 agonist)
b. Acebutolol (B1 antagonist)
*c. Lisinopril (Angiotensin Converting Enzyme Inhibitor/ACE-I)
d. Losartan (Angiotensin II Receptor Blocker/ARB)

8. Which of the following physical findings would NOT be expected in a patient with a consolidated pneumonia? (2 Points)

*a. Tympanitic sounds with percussion
b. Tactile fremitus
c. Crackles on inspiration
d. Bronchophony on auscultation

9. Which of the following is the greatest risk factor for pulmonary disease? (1 Point)

a. Family history of pulmonary fibrosis
b. Family history of atopy
c. Poor air quality
*d. Prior history of smoking
e. Prior history of autoimmune disease

10. While gathering a history of presenting illness, the patient develops severe respiratory distress and is unable to answer your questions. The most appropriate action is to: (1 Point)

a. Continue and complete the H&P
b. Rush to get the key elements
c. Ask the patient if it is ok to complete the interview
*d. Delay interview and call a code blue

Note: *Indicates the correct answer. There was a total of 15 points available on the MCQ pre- and post-test
