# Supplementary material for: Impact of educational instruction on medical student performance in simulation patient
Source: Int J Med Educ. 2022 Jun 23;13:158–70. doi: 10.5116/ijme.62a5.96bf (PMC9911140; doi:10.5116/ijme.62a5.96bf)
Supplement: Supplementary file 2 — Appendix 2. Mean performance scores by COPD and Asthma case scenarios [file ijme-13-158-S2.pdf]

Appendix 2

Mean performance scores by COPD and Asthma case scenarios

Groups	N	SPE-1 mean (SD)	SPE-2 mean (SD)	t-test t score	p-value	95% CI	
						LL	UL
COPD	32	22.25 (3.78)	23.06 (4.53)	$t_{(30)} = -0.54$	.586	−2.30	0.68
Asthma	32	22.62 (3.59)	23.93 (3.99)	$t_{(30)} = -0.97$	.336	−3.21	0.59
COPD	16	22.25 (3.78)		$t_{(30)} = -0.28$	.775	−3.03	2.29
Asthma	16	22.62 (3.59)					
COPD	16		23.06 (4.53)	$t_{(30)} = -0.57$	.566	−4.95	1.21
Asthma	16		23.93 (3.99)				

Note: SPE-1 = first simulated patient encounter; SPE-2 = second simulated patient encounter; COPD = chronic obstructive pulmonary disease; CI = confidence interval; LL = lower limit; UL = upper limit. The 95% CI is reported for the difference between the means. There was a total of 44 points available.
