# Supplementary material for: Impact of educational instruction on medical student performance in simulation patient
Source: Int J Med Educ. 2022 Jun 23;13:158–70. doi: 10.5116/ijme.62a5.96bf (PMC9911140; doi:10.5116/ijme.62a5.96bf)
Supplement: Supplementary file 3 — Appendix 3. Likert Scale student feedback questionnaire [file ijme-13-158-S3.pdf]

Appendix 3

Likert Scale student feedback questionnaire

Questions	Answers	Responses
1. The educational intervention (teaching) <u>before</u> the given patient encounter was beneficial to my learning.	a. Strongly agree	32
	b. Agree	61
	c. Neutral	24
	d. Disagree	2
	e. Strongly Disagree	4
2. The debriefing *<u>after</u>* the patient encounter was beneficial to my learning.	a. Strongly agree	54
	b. Agree	61
	c. Neutral	6
	d. Disagree	1
	e. Strongly Disagree	1
3. This experience *<u>overall</u>* enhanced my ability to apply knowledge in the clinical environment.	a. Strongly agree	74
	b. Agree	37
	c. Neutral	7
	d. Disagree	1
	e. Strongly Disagree	4
